# Integrated Sports Medicine: A First Investigation of Heart Performance in Opera Singers

**DOI:** 10.3390/jfmk7020036

**Published:** 2022-04-27

**Authors:** Marco Corsi, Goffredo Orlandi, Vittorio Bini, Laura Stefani

**Affiliations:** 1 Sports Medicine Center, University of Florence, 50121 Florence, Italy; marco.corsi@unifi.it (M.C.); gorland69@gmail.com (G.O.); 2 Department of Medicine, University of Perugia, 06123 Perugia, Italy; vittorio.bini@unipg.it

**Keywords:** myocardial performance, music, sport

## Abstract

Introduction: Opera singers are continuously subjected to cardiopulmonary exercise. The impact on cardiac performance has not been studied. Our aim was to verify the impact of singing on heart performance, particularly by the evaluation of ECG and deformation parameters as strain, rotation and twist. Methods: A population of 17 OS (opera singers) underwent a 12-lead ECG and 2D echocardiographic evaluation. A post-processing analysis of the images to obtain the deformation parameters was included. The data expressed as mean as SD were compared to a group of 15 high-level athletes (A). Results: In both groups, the ECG parameters, 2D standard systodiastolic parameters and pulmonary pressure were normal, and in the OS group—LVDd: 47 ± 2.75 mm, LVSd: 31 ± 3.38 mm, E/A: 1.08 ± 0.23, RV: 27.63 ± 3.38 mm; in the A group—LVDd: 51 ± 1.50 mm, LVSd: 32 ± 2.50 mm, E/A: 2.37 ± 0.73, RV: 25.00 ± 3.00 mm. Indexed LV mass was significantly greater in athletes, while ejection fraction (EF) results were higher in OS. Deformation parameters did not differ among the two groups, with the exclusion of GLS expressing a major value in athletes. Rotational parameters resulted in the OS group similar to the athletes. Conclusions: OS show myocardial performance as high as the athletes. The data obtained suggest a positive impact of regular training as an opera singer. Deformation parameters highlight the fitness status in this group with a specific remodeling in RV in the presence of normal PP. Classic music singing appears to have a training effect on the heart. Further studies are necessary to confirm this hypothesis.

## 1. Introduction

Opera singers (OS) perform continuous physical exercises by the vocalist movements. This represents a hemodynamic commitment, especially in the muscular and cardiopulmonary district [1]. Breathing is a fundamental aspect for opera singing and can be considered a primary aspect such as phoniatrics. Technically, during singing exercises, the adaptation of posture and activity of the abdominal muscles and diaphragm produce changes in intrathoracic pressure. The latter has a physiological impact on organs contained in the rib cage, such as the lungs and heart. During singing, the muscles of the abdomen and solar plexus are pushed outward, while the belly in the umbilical region is constantly pulled inward. It has been demonstrated that opera singers have stronger chest-wall muscles and that their hearts pump better [2]. In addition to this mechanical event, the vibrations produced by the outgoing vocal signal, which is then also heard by the singer during daily vocalizations, emitted at a certain frequency, represent a further stimulus, especially on the neurovegetative system of heart and brain. It is well known that music at frequencies between 432 and 528 Hz can have an influence on the hemodynamic parameters such as heart rhythm, especially heart-rate variability (HRV) and blood pressure [3], together with the state of well-being in general. Some experiences have explored the effects on anxiety and emotions [4]. Others have investigated the ECG electrophysiological markers of regional cardiac activity [5]. However, the potential impact on myocardial performance in terms of morphological and functional adaptation has not been studied. This article presents a hypothesis on an indirect role of the effects of singing, especially lyrical, on the cardiovascular system, particularly on morphological adaptation. Vocal exercise is a major factor resulting in mechanical/physical/physiological adaptations in opera singers but not the music itself. Considering that opera singers are involved in continuous exercise by breathing training during their education [6], the comparison with highly trained athletes seems to be important. Opera singers are engaged in enhancing the elastic explosive force of the rib cage to ensure better vocal performance; therefore, the myocardial performance could be involved with a potential adaptation. For a correct interpretation of the eventual peculiar modifications in the heart’s parameters, a control group was chosen from among highly trained athletes active in canoe sports. This study aims to verify the long-term impact of singing on the heart’s performance, as measured by echocardiographic evaluation of deformation parameters on the left and right ventricles at rest condition, assuming opera singers practicing physical exercise as athletes.

## 2. Materials and Methods

From a large group of singers in a Tuscany choir, composed of 30 subjects, a subgroup of 17 opera singers (9 female and 8 males, aged 50 years) were investigasted, on the basis of regular sing-training. All the data considered for the study (Opera Singers and Athletes) were already present in our data base, as consequence of their periodical cardiological ceck-up. on the basis of regular sing-training. They were from Italy, all Caucasian, and professional singers trained daily for at least two hours for more than five years. All participants provided informal written consent to participate in the study. The study was in accordance with the Declaration of Helsinki. An informal written consent, approved from the local ethical committee, was obtained, as is regularly performed for sports medicine and lifestyle evaluation. All the subjects were studied at rest condition. A complete anamnesis, including familiar history and potential previous diseases, was obtained. A brief story of lifestyle habits, particularly the quantity of spontaneous physical activity in terms of numbers of daily/steps or about the eventual additional programmed physical activity at moderate intensity, was investigated through a simple questionnaire (IPAQ questionnaire) [7] administered during the anamnestic report, in order to evaluate if the population investigated could be considered as active, moderate active or sedentary. Despite the IPAQ questionnaire not being a valid and reliable tool to evaluate training status in athletic populations, it has been dedicated to the evaluation of the level of sports activity or training; however, its results are fundamental in this context to verify the level of spontaneous physical activity, which represents an indicator of basic lifestyle aspects [8,9]. As the general questionnaire is dedicated (IPAQ) to investigate daily and weekly physical activity, most OS indicated that they practice less than 3 h of physical activity per week, and therefore were classified as low active or moderate active (IPAQ < 700 < 2519). An exclusion criterion was occurrence of sudden death in family history. The eventual coexistence of mild hypertension, compensated diabetes, or atypical chest pain did not represent exclusion criteria. After a standard clinical evaluation and measurement of systolic and diastolic blood pressure, they submitted to a 12-lead standard ECG and echocardiographic examination. All data were compared to a group of trained athletes practicing a high intensity sport, canoeing.

### 2.1. Echocardiographic Exam

Following the American Heart Association guidelines, [10] a complete traditional echocardiogram using an MLX8exp Release F100001 (Esaote, Florence, Italy) equipped with a 2.5 MHz probe was performed for each subject at rest. The basal 2D systodiastolic and Doppler parameters were obtained: interventricular septum (IVS), posterior wall thickness (PW), left ventricular end-diastolic diameter (LVDd), left ventricular end-systolic diameter (LVSd), left atrial dimension (LA) and volume, aortic root, dimension, peak velocities of pulsed wave Doppler transmitral flow during early diastole (E) and atrial systole (A), deceleration time of early diastolic flow (DTc), and isovolumetric relaxation time (IVRT), with addition of tissue Doppler (E1, A1, S1) parameters. The evaluation of left ventricular mass index (iLV mass g/m^2^) was obtained using the Devereux formula [11]. Ejection fraction (EF) was calculated by the Simpson rule method. The degree of severity of the eventual valvular insufficiency, described as the extent of the regurgitant jet on a 0 to 4+ scale, was assessed, if present, using the color-flow mapping method from the four-chamber view, according to ACC/AHA guidelines [12]. In addition, the left atrial volume and atria were obtained. Particular attention was directed toward the right ventricle (RV) definition, calculating the RV dimensions from the short-axis view, RVOT 1 and 2 tracts, and the RV systolic and diastolic area as expressing normal RV function. The S wave velocity by the TDI measurement of the RV chamber was also obtained. The FW RV lateral thickness was measured from the subcostal view. 

### 2.2. Strain Analysis by Speckle-Tracking Model

The 2D images of 4-chamber views were post-processed with X-Strain software to provide an angle-independent tool for the evaluation of velocities and strain. This software allows automatic evaluation of the dynamic properties of the endocardial border and of the subendocardial tissue from 2D B-mode echocardiographic clips. Strain analysis by speckle tracking is independent of translational motion, tethering effects of the nearby regions, and it thus allows uniformity of measurements through the normal LV myocardium. The endocardial border is drawn by the operator in a 4-chamber view on a single frame from one annulus to another; the first and last points delineate the mitral plane. Other acquisitions are from 2C- and 3-chamber views. The other regional segment points were automatically set by the dedicated software. The LPSS was measured in basal and mid-apical segments of the LW and IVS from the images captured at rest. The focused apical 4-chamber view was used for the LV and a modified apical 4-chamber view for the RV. In both views, frame rates were adjusted to between 40 and 90 frames per second.

**Figure 1:**
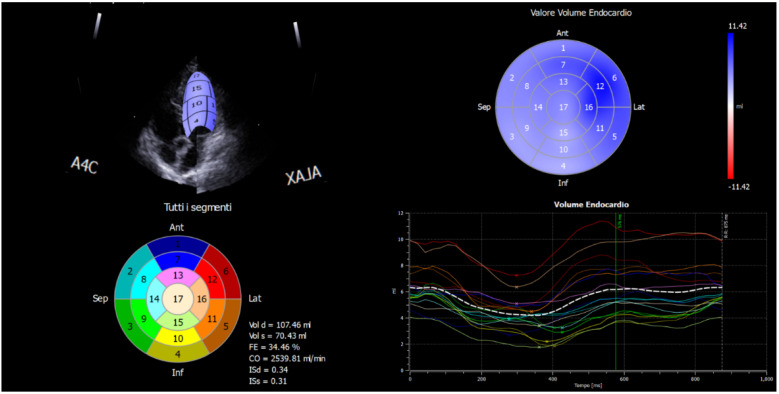
Example of strain analysis and reconstruction by X-Strain software. The single chamber deformation segments are associated with different colored lines and reported in the circle by numbers that define the exact anatomical origin. Each colour corresponds to the deformation of a single myocardial segment. The minus sign (−) on the figure corresponds to the deformation values (strain). A4C = Global longitudinal strain 4 chambers; ALAX = Global longitudinal strain 3~chambers; Ant = Anterior wall; Lat = Lateral wall; Inf = Inferior wall; Sep = Septal wall.

During the offline analysis (Echo Pac, Version 6.0, GE Healthcare, Horten, Norway) a region of interest was placed around the LV from the basal septum through to the basal lateral wall, ensuring that the entire myocardium was encompassed within. This provided six myocardial segments and an average of these provided a global index of LV longitudinal ε. For the RV, the offline analysis involved placing the region of interest around the RV lateral wall only from base to apex. From the short-axis view, captured at basal and apex segments of the LV chamber, the rotation was calculated. The net difference of the basal vs. apex rotation values gave the torsion. Technically, from that window, a short-axis image of the LV apex, as circular as possible and just proximal to the level with end-systolic LV luminal obliteration, was obtained by angulation of the transducer. The short-axis views, captured at the mitral valve plane and apex level, were later processed by the speckle-tracking X-Strain software. The software asks the operator to provide the initial position of the tracking points. [13]. An example of the final reconstruction of the post-processing analysis is reported in Figure 1.

### 2.3. Statistical Analysis 

Normality of variables was checked by the Shapiro–Wilk test. Because of their asymmetric distribution, all data are reported as median and quartile deviation. The comparisons between LPSS values of basal and mid-apical segments for each group were performed using the Mann–Whitney U test (SPSS Statistics 13, IBM, California State University Channel Islands, Camarillo, CA, USA). Two-sided probability value (*p*) of 0.05 was considered statistically significant. 

#### ECG Parameters 

All baseline electrocardiograms, obtained at rest conditions, were reviewed by two certified cardiologists. 

ECGs were analyzed according to the Minnesota code for resting electrocardiograms [11], consisting of nine domains: the presence of a Q wave, QRS axis deviation, high-amplitude R waves, ST segment depression, T wave abnormalities, A–V conduction defects, ventricular conduction defects, arrhythmias and a miscellaneous items domain (including low QRS amplitude, ST segment elevation, pathologic QRS transition zone and high P or T wave). The Q–T interval was also manually assessed and corrected according to the Bazett formula (QTc = QT time/√RR interval) [14]. The QT interval was measured in a lead free of noise and arrhythmic beats. QTc ≥ 440 ms was classified as prolonged. An ECG was positive for CAD if Q wave, ST segment depression and/or a pathologic T wave was present [12]. All ECG features were analyzed only if the ECG was considered to be of sufficient quality to be interpreted. 

## 3. Results

All the subjects included in the study were healthy. None had diabetes or symptoms compatible with coronary artery disease (CAD). None from either the OS or A group had a positive anamnesis for sudden death or symptomatic cardiovascular disease. Particularly, all were negative for hypertension or diabetes. During the IPAQ investigation, most OS declared having practiced less than 3 h of physical activity per week and were therefore classified as low active or moderate active (IPAQ < 700 < 2519) [7]. On the contrary, at the time of anamnesis the athletes regularly trained seasonally at high intensity. The IPAQ investigation of the canoe athletes confirmed that they were active (IPAQ > 2519). As reported in the tables, all data were expressed as median and quartile deviation and in the range of normality for both. Significant (*p* < 0.05) and very significant (*p* < 0.001) differences were observed in the two populations for several of the parameters considered. All data obtained were within the normal range. As expected, the athletes showed a significantly higher dimension of the LV chamber (LVDd A: 51 ± 1.50 mm vs. LVDd OS: 47 ± 2.75 mm; *p* < 0.04) with a greater iLV mass (iLV mass A 108.79 ± 11.36 g/m^2^ vs. CMI OS 81.05 ± 12.51 g/m^2^), while EF was lower (*p* < 0.027) when compared to OS (Table 1). The right atrial volume was higher in athletes (A: 38 ± 11.50 mm vs. OS 23 ± 6.38; *p* < 0.024) (Table 1). This aspect could be initially interpreted as an expression of the continuous physical training impact on the heart, inducing a constant venous return, as in the case of canoe sports activity. Diastolic parameters were normal in the two groups; however, for the athletes this function resulted in a better comparison with the OS, particularly for the LV TDI parameters (Table 1). This aspect can be interpreted as an age-related physiological pattern among the subjects investigated. The two groups differed in age and for the time of regular aerobic exercise. These differences may have a role in the data obtained. The age-related and training-related changes in these two populations are potential aspects to address when considering the similarity of differentiation in myocardial adaptation (e.g., right/left ventricle adaptations, as presented in Table 1). 

Regarding the RV chamber, the FW thickness was found increased in OS, despite the presence of normal morphology and dimension (Table 1). This is the principal original aspect of this pilot study. Pulmonary pressure (Table 1), in particular, was higher in athletes (A PP: 10 ± 6.05 mmHg) compared to OS (OS PP: 8.18 ± 1.09 mmHg). This peculiar aspect could be initially referred to a specific effect of the music physical exercise on OS, suggesting an RV myocardial remodeling not associated with hemodynamic impact on the pulmonary circulation. 

According to these results, strain parameters (GLS from 2C, 3C, 4C chamber view) were also normal in the left and right ventricle chambers, with major and significantly negative values in athletes (Table 2). The rotation and twist movements (Table 2) did not show any significant differences. In addition to the evidence of a normal function of both the RV and LV in the two groups, the data support the hypothesis of a physiological myocardial behavior in OS as well as in athletes, despite experiencing different kinds of exercise. It appears that the longitudinal deformation, GLS%, is represented more in canoe athletes whose LV dimensions are also greater. On the contrary, the circumferential and rotation contribution to fiber contraction in this sample is similar and a potential expression of pressure intracardiac loading in OS.

All the ECG parameters were normal for both groups, excluding HR, which was significantly lower in athletes as consequence of a major training. No abnormal data potentially related to eventual arrhythmic acute events were found (Table 3).

## 4. Conclusions 

According to the literature, music has several beneficial effects, especially on heart hemodynamics. Studies show benefits on several cardiac aspects such as BP and HR variability [1,15,16], although these studies were conducted on listening to music at different frequencies. The impact of music on myocardial morphology and remodeling among singers, however, has not been explored. Few data are on the contrary, available in the context of physical performance in opera singers, especially when the music is not exclusively listened to but also produced. The question is therefore if opera singing can be considered fitness, starting from an initial study that had, however, exclusively investigated the body composition [17]. Opera singers are not usually evaluated by specific sports medicine texts and this is the first study that highlights the importance to evaluate cardiac performance in terms of morphological adaptation. The study is therefore based on the hypothesis to investigate, initially, OS from the morphological heart’s remodeling, as in the case of athletes, considering their vocal exercise as a physical one, and to compare OS myocardial morphology to very high-intensity trained athletes. To evaluate and verify the long-term impact to potential long-term myocardial adaptation, this investigation was conducted at rest condition, which is the best situation to study eventual subtle modification of myocardial morphology and contraction. The groups investigated were both trained, in their context, constantly for several years. For this reason, specific measurement of deformation parameters was applied to provide an in-depth study of myocardial performance. No additional information in this context could be obtained in the case of exercise tests, where many other hemodynamic components of cardiac response to exercise could play a confounding role in its correct interpretation. Therefore, no exercise tests were included in the protocol study. This approach can be considered as the best model presently, considering this is the first study reported in the literature that is dedicated to this evaluation. The data obtained, particularly for the RV chamber, highlight the importance for investigating this category. The actual interpretation of the results confirms the normal function of the heart in absence of any negative impact on the heart. A specific involvement of the RV seems to be evident. In addition, any change in chest volume can support the development of muscle strength to overcome the increased lung retraction and rib cage expansion force. In this context, it is important to train the management of the body, to improve posture control to maintain a normal heart’s performance. 

The correct interpretation of this aspect, without any implication on the pulmonary pressure, will need a larger investigation. 

The eventual ECG abnormal pattern has also been explored in parallel. The data obtained showed that the OS have a high myocardial performance level, similar to the athletes. Despite OS being substantially less active, the echocardiographic values such as deformation parameters (twist and rotation) are comparable to the athlete group. This is the first study reported in the literature that evaluates singing practice as physical exercise. The presence of significantly higher values of FW thickness in OS with a normal pulmonary pressure is particularly relevant. This aspect can be considered in agreement with the physiological effects of constant exercise in which an artificial air column is produced. The air column acts as a sounding board for producing the voice and creates a type of slight overload in the right ventricle sections. In addition, the right venous return seems to be unaffected by this situation according to the low involvement of the right atrium volume. Therefore, the data support the positive effect of the music exercise in both heart ventricles. Furthermore, pathological ECG abnormalities were not found. Currently, only one study [18] reports an indirect qualitative investigation into the characteristics and effects of music when accompanying exercise. No studies have explored the eventual implications of direct myocardial impact of music exercise on morphological remodeling. 

The present study provides an initial effort to integrate subjects not yet included in sports medicine evaluation. Despite this incomplete approach in sports medicine, especially of the recent transversal interest of the sports medicine to a large population of subjects at the limit of effective training, this pilot study can be applied as a stimulus to expand this point of view. Further studies will be necessary to confirm these data and to explore in more depth the different effects of music exercise. 

## 5. Limits 

This study is mainly limited owing to the small sample investigated; therefore, it should be considered a pilot study. Another limitation in the investigation is the absence of differences by gender. Some other aspects will need to be investigated, especially those related to long-term exposure to lyrica singing in the ergometric and spirometric exercise performance. 

## Figures and Tables

**Table 1:** General and 2D-echocardiographic parameters of left and right chambers.

	Opera Singers		Athletes		*p*
	Median	Quartile Deviation	Median	Quartile Deviation	
Age (y)	55	9.75	17	2	<0.001
BMI (kg/m^2^)	26.08	4.52	23.04	1.41	0.004
LVDd (mm)	47	2.75	51	1.50	0.004
LVSd (mm)	31	3.38	32	2.50	0.486
LV IVS (mm)	9.40	0.89	9.70	0.50	0.190
LV PW (mm)	9.20	0.66	9.60	0.50	0.887
EF%	66.50	5.88	62.00	2.50	0.027
iLV mass (g/m^2^)	81.05	12.51	108.79	11.36	<0.001
MAPSE (mm)	17.50	1.38	18	1.50	0.180
E/A	1.08	0.23	2.37	0.73	<0.001
DTc (ms)	211.50	20.25	197	35	0.429
E1 (cm/s)	9	1.56	15	1.50	<0.001
A1 (cm/s)	9.70	0.98	5	1	<0.001
RV (mm)					
RVOT Prox	27	3.38	25	3	0.372
RVOT distal	24	2.25	20	3.50	0.062
RV basal	32.50	4.25	35	2.50	0.026
RV medium	25	3.25	28	3.50	0.041
RV long	66	8.13	71	5.50	0.029
RV Free Wall	7	0.96	4.40	0.65	<0.001
PP (mmHg)	8.18	1.09	10	6.05	0.025
RV (Area T) (mm^2^)	16.54	3.16	25	2.50	<0.001
RV (Area S) (mm^2^)	8.36	1.69	13	2.50	<0.001
RV TDI S’ (cm/s)	12	0.50	13	2	0.005
Aortic Root (mm)	24	3.88	30	2	0.006
LA Volume (mL)	31	7.25	33	3	0.552
RA Volume (mL)	23	6.38	38	11.50	0.024

Data are expressed as median and quartile deviation. Bold font indicates *p* < 0.05. LVDd: left ventricle diastolic diameter; LVDd: left ventricle diastolic diameter; LV IVS: left ventricle interventricular septum; LV PW: left ventricle posterior wall; EF: ejection fraction; iLV mass: indexed left ventricle mass; RV: right ventricle; RVOT: right ventricle outflow tract; PP: pulmonary pression; LA: left atrium; RA: right atrium.

**Table 2:** Echocardiographic strain and twist parameters of opera singers and athletes.

	Opera Singers		Athletes		*p*
	Median	Quartile Deviation	Median	Quartile Deviation	
4D Global Strain (%)	−17.71	2.74	−23.30	2.36	0.003
GLS4c (%)	−18.74	2.95	−20.88	2.60	0.097
GLS3c (%)	−16.33	4.17	−21.46	2.96	0.009
GLS2c (%)	−15.39	3.95	−21.22	2.24	0.002
RV Strain (%)	−18.48	3.35	−23.30	−2.36	0.002
Apical Rotation	3.24	2.13	4.60	0.95	0.199
Basal Rotation	−4.63	1.68	−4.43	0.42	0.968
Twist	7.49	2.32	8.50	1.10	0.502

Data are expressed as median and quartile deviation. Bold font indicates *p* < 0.05. GLS: global longitudinal strain.

**Table 3:** ECG parameters of opera singers and athletes.

	Opera Singers		Athletes		*p*
	Median	Quartile Deviation	Median	Quartile Deviation	
HR	71.50	6.38	61	5.50	0.010
P	101	17	85	16	0.177
PR	158	20.75	156	14	0.890
QRS	91	6	100	9	0.065
QTc	416	16.50	418	22	0.635

Data are expressed as median and quartile deviation. Bold font indicates p < 0.05. HR: heart rate; QTc: corrected QT interval with Bazett formula.

## Data Availability

Data supporting reported results can be found in archived datasets in Sports Medicine Center-AOUC (Azienda Ospedaliero Universitaria Careggi), University of Florence.
